# Spirometric assessment of emphysema presence and severity as measured by quantitative CT and CT-based radiomics in COPD

**DOI:** 10.1186/s12931-019-1049-3

**Published:** 2019-05-23

**Authors:** Mariaelena Occhipinti, Matteo Paoletti, Brian J. Bartholmai, Srinivasan Rajagopalan, Ronald A. Karwoski, Cosimo Nardi, Riccardo Inchingolo, Anna R. Larici, Gianna Camiciottoli, Federico Lavorini, Stefano Colagrande, Vito Brusasco, Massimo Pistolesi

**Affiliations:** 1 Section of Respiratory Medicine, Department of Experimental and Clinical Medicine, Careggi University Hospital, University of Florence, Largo A Brambilla 3, 50134 Florence, Italy; 2 Division of Radiology, Mayo Clinic Rochester, 200 1st St SW, Rochester, MN, 55902 USA; 3 Department of Physiology and Biomedical Engineering, Mayo Clinic Rochester, 321 3rd Ave SW, Rochester, MN, 55902 USA; 4 Section of Radiodiagnostic, Department of Experimental and Clinical Biomedical Sciences, University of Florence, Largo A Brambilla 3, 50134 Florence, Italy; 5 Section of Pulmonology, Department of Cardiovascular and Thoracic Sciences, Fondazione Policlinico Universitario A. Gemelli IRCCS, Largo F. Vito 1, 00168 Rome, Italy; 6 Department of Radiological Sciences, Fondazione Policlinico Universitario A. Gemelli IRCCS, Largo F. Vito 1, 00168 Rome, Italy; 7 School of Medical and Pharmaceutical Sciences, University of Genoa, Via Balbi, 5, 16126 Genoa, Italy

**Keywords:** Pulmonary emphysema, COPD, Small airway disease, Respiratory function tests, Spirometry, Tomography, Radiomics, Area under curve

## Abstract

**Background:**

The mechanisms underlying airflow obstruction in COPD cannot be distinguished by standard spirometry. We ascertain whether mathematical modeling of airway biomechanical properties, as assessed from spirometry, could provide estimates of emphysema presence and severity, as quantified by computed tomography (CT) metrics and CT-based radiomics.

**Methods:**

We quantified presence and severity of emphysema by standard CT metrics (VIDA) and co-registration analysis (ImbioLDA) of inspiratory-expiratory CT in 194 COPD patients who underwent pulmonary function testing. According to percentages of low attenuation area below − 950 Hounsfield Units (%LAA_-950insp_) patients were classified as having no emphysema (NE) with %LAA_-950insp_ < 6, moderate emphysema (ME) with %LAA_-950insp_ ≥ 6 and < 14, and severe emphysema (SE) with %LAA_-950insp_ ≥ 14. We also obtained stratified clusters of emphysema CT features by an automated unsupervised radiomics approach (CALIPER). An emphysema severity index (ESI), derived from mathematical modeling of the maximum expiratory flow-volume curve descending limb, was compared with pulmonary function data and the three CT classifications of emphysema presence and severity as derived from CT metrics and radiomics.

**Results:**

ESI mean values and pulmonary function data differed significantly in the subgroups with different emphysema degree classified by VIDA, ImbioLDA and CALIPER (*p* < 0.001 by ANOVA). ESI differentiated NE from ME/SE CT-classified patients (sensitivity 0.80, specificity 0.85, AUC 0.86) and SE from ME CT-classified patients (sensitivity 0.82, specificity 0.87, AUC 0.88).

**Conclusions:**

Presence and severity of emphysema in patients with COPD, as quantified by CT metrics and radiomics can be estimated by mathematical modeling of airway function as derived from standard spirometry.

**Electronic supplementary material:**

The online version of this article (10.1186/s12931-019-1049-3) contains supplementary material, which is available to authorized users.

## Background

Chronic obstructive pulmonary disease (COPD) is a complex condition with a wide spectrum of clinical presentations and pathological features unified under the spirometric definition of airflow obstruction. Airway narrowing and parenchymal destruction are recognized as the mechanisms responsible for airflow obstruction in COPD, but they cannot be distinguished by standard spirometry. However, standard spirometry is one of the most employed variables for patients enrollment and outcome evaluation in clinical and pharmacologic studies.

In recent years, chest computed tomography (CT) allows to depict and measure in vivo the lung pathologic changes of COPD by quantifying parenchymal destruction, the direct sign of emphysema, as well as bronchial wall thickening and gas trapping, which represent direct and indirect signs of conductive airway disease, respectively [1, 2]. A closer imaging definition of whether conductive airway disease or emphysema is the predominant mechanism of airflow obstruction has been lately obtained by using co-registration analysis of inspiratory and expiratory CT scans [3]. Nowadays more information is extracted from imaging data using advanced feature analysis representing what is called “radiomics” [4]. Artificial neural networks and statistical models are available to provide radiologists and clinicians with objective and reproducible computer-based evaluations of lung parenchyma. In particular, CALIPER (Computer Aided Lung Informatics for Pathology Evaluation and Rating) recently developed at Mayo Clinic (Rochester, MN), is a computational platform for the near real-time characterization and quantification of lung parenchymal patterns on CT scan [5, 6].

Altogether, quantitative and qualitative studies have shown that CT can allow distinguishing not only between airway and parenchymal abnormalities, but also between subtypes of emphysema, i.e. centrilobular, panlobular, and paraseptal [7]. However, a widespread routine use of CT for the assessment of COPD in clinical practice and clinical and pharmacologic studies cannot be currently foreseen due to radiation exposure and limited instrumental availability in the face of the COPD rapidly increasing prevalence of the disease [8].

In a previous study we have shown that a probabilistic model based on body mass index, FEV_1_ as percent of predicted, FEV_1_/VC and DLco as percent of predicted could be used to estimate emphysema quantified on CT [9]. Limitations of that approach for clinical practice or clinical and pharmacologic trials are that DLco is not always available and that standard pulmonary function parameters have a wide inter-individual variability, even after normalization for ethnicity, age, and body size [10]. Furthermore, probabilistic models rely on regression coefficients that reflect the characteristics of the training set. These limitations may be possibly overcome by mathematical models studying directly the biomechanical characteristics of airway function of each patient [11].

The aim of the present study was to assess whether a mathematical model designed to fit the shape of the maximum expiratory flow-volume curve (MEFV) obtained by standard spirometry could provide estimates of the presence and the severity of emphysema comparable with parameters used to assess emphysema extent derived from quantitative CT and CT-based radiomics.

## Methods

This prospective two-center study was approved by the institutional Ethics Committees of the University of Florence and of the Catholic University of Sacred Heart in Rome. The study is based on a retrospective interpretation of prospectively acquired data. From January 2012 to December 2016, subjects with diagnosis of COPD (post-bronchodilator FEV_1_/VC < 0.70) [12, 13] were considered for inclusion if they satisfied the following inclusion criteria: age 40–85 years, smoking history > 10 pack-years, no COPD exacerbations within 1 month, no diagnosis of cardiac disease, and acceptance to participate by written informed consent. Patients with a reversible airflow obstruction greater than 12% and 200 ml after inhalation of bronchodilator (according to ATS recommendations) or those with a clinical history of present or previous asthma episodes were excluded [12, 14]. Thirty-eight out of 232 eligible subjects were excluded because of incomplete data or coexisting abnormalities on CT scan.

### Functional evaluation

Subjects underwent complete pulmonary function evaluation by using a mass-flow sensor and multigas analyser (V6200 Autobox Body Plethysmograph Sensor Medics, Yorba Linda, CA, USA, or Platinum Elite™ Body Plethysmograph, Medical Graphics Corporation, St. Paul, MN, USA), arterial blood gases by Radiometer ABL90 FLEX or ABL800 FLEX (Brønshøj, Denmark). Pre- and post-bronchodilator spirometry, lung volumes, and single-breath DLco were obtained according to standard ATS/ERS (American Thoracic Society/European Respiratory Society) recommendations [15].

### CT scanning technique and analysis

**Fig. 1:**
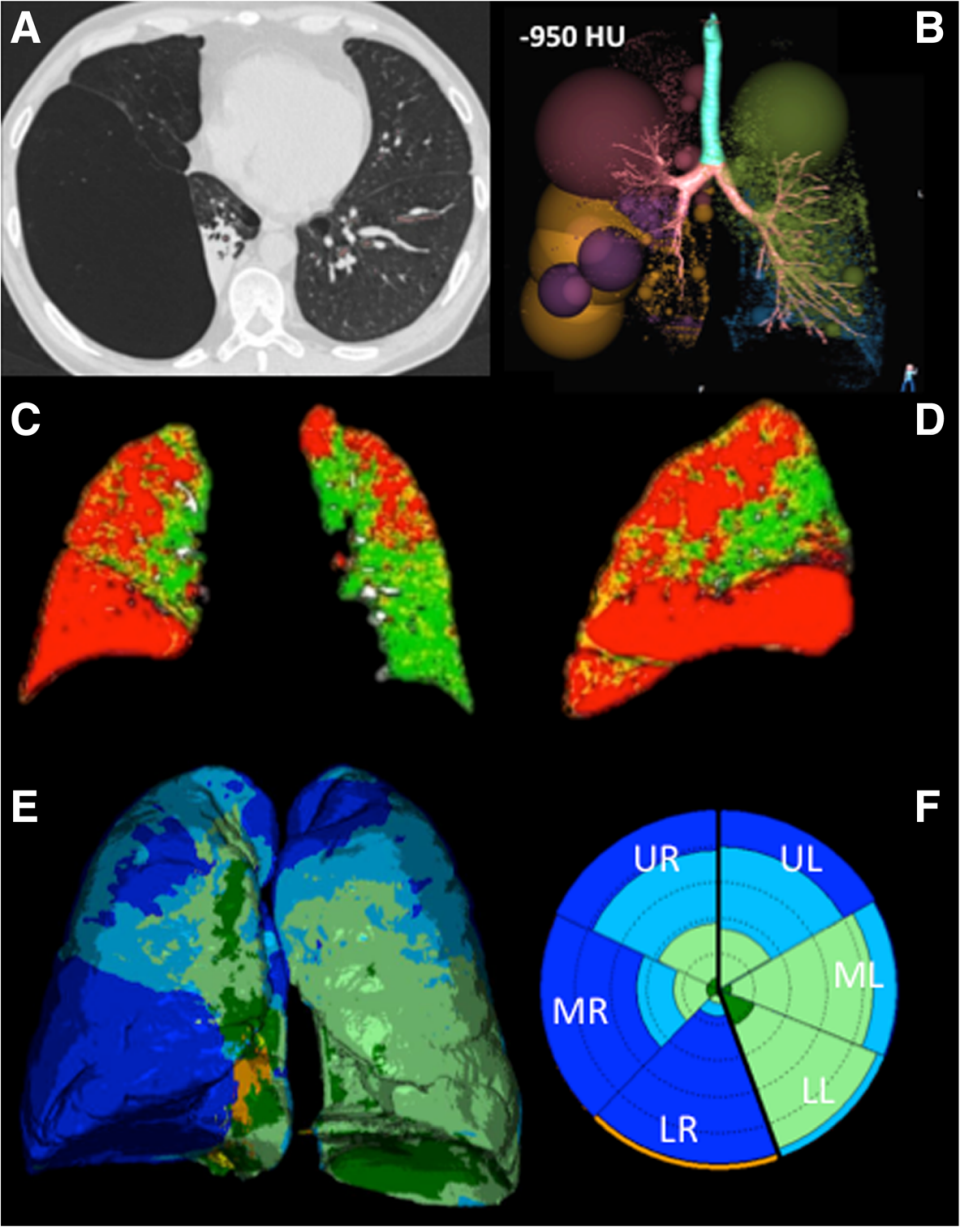
Lung parenchyma representations at CT scan after post-processing with different software programs in a patient with severe emphysema. **a**. Axial CT scan shows advanced destructive emphysema with a giant bulla in the right lower lobe adjacent to an area of passive atelectasis. **b**. Volume rendering of the densitometric analysis performed by VIDA shows the location and severity of emphysema at inspiratory scan (threshold -950HU) displaying spheres whose diameter is proportional to the relative volume of emphysema in each region. **c-d**. Coronal and Sagittal 2D images obtained by co-registration of inspiratory and expiratory CT scans by Imbio LDA show the location of emphysema (*red*), functional airways gas trapping (*yellow*), and normal lung (*green*). **e**. Volume rendering of the lung texture analysis performed by CALIPER shows the 3D distribution of the different lung patterns, including Normal (*dark green*), Mild Low Attenuation Area (LAA, *light green*), Moderate LAA (*light blue*), Severe LAA (*dark blue*), Ground-glass (*yellow*), Reticular (*orange*). The glyph **f** provided by CALIPER summarizes the location and amount of the different lung patterns. The overall area of the glyph represents the computed total lung volume, the partitions with thick radial lines illustrate the relative volumes of the left (L) and right (R) lungs, which are further divided with thin radial lines into three regions, each representing the upper (U), middle (M), lower (L) lung zones. In this patient severe LAA dominates in the right lower and middle lung zones, whereas middle and lower left zones are characterized by mild and moderate LAA

In each center volumetric chest CT scans were obtained by the same team and the same CT scanner (SOMATOM Sensation 64 or SOMATOM Definition FLASH 128, Siemens, Erlangen, Germany) within 48 h of the functional evaluation. CT scans were acquired at full inspiration and forced end-expiration using the acquisition protocol adopted in the COPDGene Study [16] with the following parameters: 120 kVp, 200 mAs, rotation time 0.5 s, slice thickness 0.6 mm, pitch 1.1 and reconstructions kernels b31f (smooth) and b70f (sharp). The same personnel carefully trained all subjects before undergoing CT scans for consistency in CT data acquisition. Subjects were instructed on how to perform respiratory maneuvers while lying supine in the CT scanner with arms fully abducted. No contrast medium was injected. Post-processing image analysis was performed on images with reconstruction kernel b31f by using three different software programs: VIDA, Imbio LDA, CALIPER (Fig. 1a-f).

- *VIDA analysis* (Fig. 1b). We used the Pulmonary Workstation Apollo 2.1 (VIDA, Coralville, IA, USA) installed onsite to segment airways and lungs and to calculate the relative volumes of lung attenuation area with values below − 950 Hounsfield Units (HU) at inspiration (%LAA_-950insp_) and below -856HU at expiration (%LAA_-856exp_). Thresholds at − 950 HU and -856HU were chosen as densitometric cut-offs for emphysema and total gas trapping, respectively [1, 17, 18]. The analysis was fully automated with the possibility to correct manually any mistake in airway or lobe segmentation. Bronchial wall thickening was calculated as airway wall thickness at an internal perimeter of 10 mm (AWTPi10) [19].
- *Imbio LDA analysis* (Fig. 1c-d). Co-registration analysis performed by Imbio LDA (Minneapolis, MN, US) automatically pairs inspiratory and expiratory CT scans to provide percentages of normal lung (percentage of voxels with CT attenuation greater than − 950 HU at inspiration and greater than -856HU at expiration), persistent low density area (%pLDA, voxels with CT attenuation below -950HU at inspiration and below -856HU at expiration), and functional low density area (%fLDA, voxels with CT attenuation above -950HU at inspiration and below -856HU at expiration) [3]. %fLDA represents the non-emphysematous contribution to total gas trapping, namely the fraction of gas trapped at end expiration because of airway closure or extreme flow limitation. Moreover, Imbio LDA provides parametric response maps showing the regional distribution of each lung pattern. The analysis was fully automated and performed on an online platform called Imbio Launchpad.
- *CALIPER analysis* (Fig. 1e-f). Inspiratory CT scans were post-processed by using CALIPER (Mayo Clinic, Rochester, MN, USA), a computational platform for the near real-time characterization and quantification of seven lung parenchymal patterns on CT scans, including Normal, Mild Low Attenuation Area (LAA), Moderate LAA, Severe LAA, Ground-glass, Reticular, and Honeycombing (Fig. 1e) [5]. CALIPER is based on histogram signature mapping techniques trained through expert radiologist consensus assessment of pathologically confirmed datasets obtained through the Lung Tissue Research Consortium [6, 20]. For each subject, CALIPER outputs a glyph similar to a radial space-filling plot providing an iconic summary of the volumetric parenchymal classification, thus facilitating the comprehension of the multidimensional source data [6]. The area of the glyph represents the computed total lung volume. The glyph is partitioned with radial lines to illustrate the relative volumes of the left and right lungs and further divided into three regions, each representing the upper/middle/lower lung zones (Fig. 1f). CALIPER analysis was fully automated and was performed onsite in the laboratory.

### Emphysema Severity Index (ESI)

ESI is based on a parametric biomechanical model representing a theoretical approximation of the shape of the descending limb of the MEFV curve computed by assuming that at a given time the pressure lost by a fluid flow conveyed in a cylindrical duct is inversely related to its diameter and directly related to the fluid specific friction factor, density, and velocity (see the Additional file 1 for theoretical and mathematical details). The computation does not require standardization of input parameters, as it is directly related to the shape of the curve. Therefore, ESI is independent from percentage predicted values of pulmonary function variables. ESI value was computed in each patient using a specifically developed software application, whose theoretical basis is reported on the online supplement. A numerical output value ranging from 0 to 10 was used to stratify the dataset of 194 COPD patients according to the estimated emphysema severity.

### Data analysis and statistics

The relationship between ESI scores, functional data and CT metrics was assessed by Pearson correlation coefficients. Robust Steiger’s Z-test was used to evaluate the statistical significance of differences in correlations between CT-metrics relative to emphysema (%LAA_-950insp_ and %pLDA) and ESI, FEV_1_%, and FEV_1_/FVC.

By using the two thresholds of %LAA_-950insp_ reported in literature to define absence of significant emphysema (6%) [7] and severe emphysema (14%) [21] we classified the patients in three subgroups: no emphysema (NE, %LAA_-950insp_ < 6), moderate emphysema (ME, 6 ≤ %LAA_-950insp_ < 14), and severe emphysema (SE, %LAA_-950insp_ ≥ 14).

A pairwise dissimilarity matrix was derived using SILA (**S**cale **I**ndicative of **L**ung parenchyma **A**bnormality). SILA between a pair of CALIPER quantified CT lung volumes was computed as a cumulative aggregate of the differentials of normalized distributions of ordered (as mild, moderate, and severe) CALIPER exemplars. The unique clusters representing similar groups of patients were identified by unsupervised clustering of the 194 × 194 dissimilarity matrix using affinity propagation. The method does not require an a priori specification of the number of desired clusters.

Chi-squared test was used to determine any significant differences between expected and observed frequencies in the clusters obtained by CALIPER and the three groups defined on the basis of %LAA_-950insp_ ranges (NE, ME, SE).

Analysis of variance, Welch’s t, and Games-Howell post-hoc tests were used to evaluate differences of the mean values of pulmonary function tests and ESI mean values among the three groups of emphysema severity defined by VIDA and Imbio LDA, as well as the clusters obtained by CALIPER.

To evaluate the performances of the ESI software as a classification tool we used logistic regression analysis. In each patient we estimated the probability of being affected by “severe” emphysema (%LAA_-950insp_ ≥ 14, data computed by VIDA) given the ESI value obtained by spirometry. In a similar manner we estimated the probability of “absence” of significant emphysema (%LAA_-950insp_ < 6, data computed by VIDA) given the ESI value.

A ten-fold cross validation was performed over the entire dataset and we calculated the True Positives and False Positives rates for each fold. Sensitivity, specificity and AUC were evaluated by ROC curve analysis. In particular we estimated in each patient the probability of being affected by “severe” emphysema (%LAA_-950insp_  ≥14, data provided by VIDA) given the value of ESI score obtained by spirometry; π = Pr (Y = “Severe” | X = ESI) and the probability of “absence” of emphysema (%LAA_-950insp_ < 6, data provided by VIDA) given the value of ESI score obtained by spirometry; π = Pr (Y = “Absence” | X = ESI).

The software programs included Mathcad (version 2001; Mathsoft), SPSS/PC WIN 11.5.1 (SPSS, Chicago, IL), C++ programming language, and Orange [22]. Values of *p* lower than 0.05 indicated statistical significance. Data are expressed as mean and standard deviation (SD).

## Results

Table 1 describes anthropometric, pulmonary function, and CT metrics data of the 194 subjects included in the study. Subjects were distributed across all GOLD stages: 55 stage I, 62 stage II, 56 stage III, and 21 stage IV.

**Table 1:** Anthropometric, pulmonary function and CT metrics data of the 194 COPD subjects included in the study

Sex (M:F)	154:40	
Age (yr)	70 (8.0)	
BMI (kg/m^2^)	27 (4.6)	
Smoking history (pack-years)	52 (27)	
FEV_1_ (% pred)	63 (26)	
FEV_1_/VC	48 (13)	
FEV_1_/FVC	52 (13)	
TLC (% pred)	108 (17)	
DLco (% pred)	69 (24)	
RV (% pred)	137 (47)	
FRC (% pred)	130 (33)	
RV/TLC	50 (14)	
VIDA	%LAA_-950insp_	14 (12)
	%LAA_-856exp_	45 (20)
Imbio LDA	%pLDA	12.2 (12.5)
	%fLDA	37.1 (14.0)
	% Normal	49.1 (21.1)

Data are expressed as mean (SD). Legend: *BMI* body mass index, *DLco* diffusing lung capacity for carbon monoxide, *FEV*_*1*_*%* forced expiratory volume in 1 s, *%fLDA* percentage of functional low density area, *FRC* functional residual capacity, *FVC* forced vital capacity, *%LAA*_*-950insp*_ percentage of lung attenuation area with values <− 950 Hounsfield Units at inspiratory CT scan, *%LAA*_*-856exp*_ percentage of lung attenuation area with values <−856 Hounsfield Units at expiratory CT scan, *% Normal* percentage of normal lung, *%pLDA* percentage of persistent low density area, *%pred* percentage of predicted, *RV* residual volume, *TLC* total lung capacity, *VC* vital capacity

Figure 1 shows CT images of a patient with advanced destructive emphysema before (Fig. 1a) and after post-processing image analysis by using the three different software programs: VIDA (Fig. 1b), Imbio LDA (Fig. 1c-d), and CALIPER (Fig. 1e-f).

**Fig. 2:**
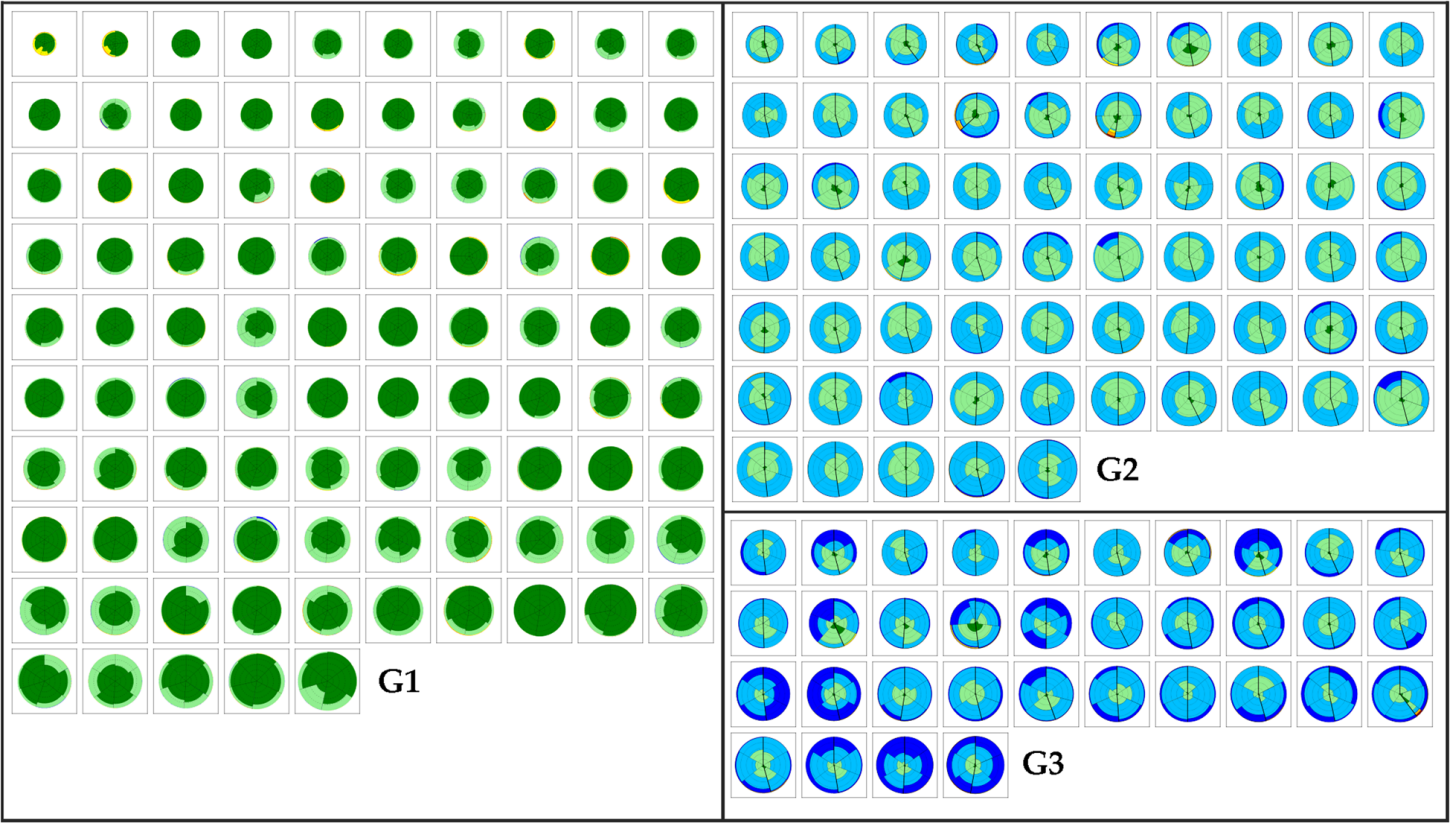
The three clusters of COPD patients stratified represented as glyphs. Clusters (G1 to G3) were the result of quantitative unsupervised clustering based on a dissimilarity matrix that captures the distribution of classified parenchymal patterns recognized by CALIPER. G1 was characterized by predominant Normal (*dark green*) and Mild LAA (*light green*) patterns, whereas G2 by predominant Moderate LAA (*light blue*) pattern and G3 by predominant Severe (*dark blue*) and Moderate LAA (*light blue*) patterns

Figure 2 illustrates the patients subdivision according to the pairwise dissimilarity matrix that identified three clusters (G1-G3), based on the SILA metric derived from the features extracted by CALIPER. Cluster G1 consisted of 95/194 (49%) subjects, G2 of 65/194 (33.5%) subjects, and G3 of 34/194 (17.5%) subjects. Across the three different clusters patients lungs were represented by a glyph, illustrating the regional composition of classified lung volume with color-coded sections proportional to the percentage of lung patterns within the region (Fig. 1f). G1 was characterized by predominant Normal and Mild LAA patterns, G2 by predominant Moderate LAA pattern, and G3 by predominant Severe and Moderate LAA patterns. Patients clustered as G1 by CALIPER had either NE or ME at VIDA, whereas patients clustered as G2 had ME or SE and all patients clustered as G3 had SE.

Table 2 shows the correlations between ESI values and functional and radiological data. The strongest correlations were seen between ESI and FEV_1_/FVC (*r* = − 0.87) and between ESI and %LAA_-950insp_*(r* = 0.81) or %pLDA (*r* = 0.80). The correlation between ESI values and AWTPi10 was not significant (*r* = 0.13). Table 3 shows the correlation between CT metrics and ESI, FEV_1_ and FEV_1_/FVC. CT metrics of emphysema (%LAA_-950insp_ and %pLDA) correlated more strongly with ESI (*r* = 0.81 and 0.80) than with FEV_1_ (*r* = − 0.50 and − 0.52) and FEV_1_/FVC (*r* = − 0.67 and − 0.68). Differences in r values were statistically significant (*p* < .001).

**Table 2:** Pearson r correlations between ESI scores and functional and radiological data

	ESI	
	*r*	*p*
%LAA_-950insp_	0.81	<.01
%LAA_-856exp_	0.74	<.01
%fLDA	0.46	<.01
%pLDA	0.80	<.01
AWTPi10	0.13	.09
FEV_1_ (%pred.)	−0.74	<.01
FVC (%pred)	− 0.30	<.01
FEV_1_/FVC (%)	−0.87	<.01
FRC (% pred)	0.69	<.01
RV (% pred)	0.65	<.01
TLC (% pred)	0.37	<.01
DLco (% pred)	−0.56	<.01

Legend: *AWTPi10* airway wall thickness at an internal perimeter of 10 mm, *DLco* diffusing lung capacity for carbon monoxide, *FEV*_*1*_ forced expiratory volume in 1 s, *%fLDA* percentage of functional low density area, *FRC* forced respiratory capacity, *FVC* forced vital capacity, *%LAA*_*-950insp*_ percentage of lung attenuation area with values <− 950 Hounsfield Units at inspiratory CT scan, *%LAA*_*-856exp*_ percentage of lung attenuation area with values <− 856 Hounsfield Units at expiratory CT scan, *%pLDA* percentage of persistent low density area, *%pred* percentage of predicted, *RV* residual volume, *TLC* total lung capacity

**Table 3:** Pearson r correlations between CT metrics and FEV_1_, FEV_1_/FVC, ESI scores

	FEV_1_/FVC		FEV_1_%		ESI	
	*r*	*p*	*r*	*p*	*r*	*p*
%LAA_-950insp_	−0.67	<.01	−0.50	<.01	0.81	<.01
%LAA_-856exp_	−0.71	<.01	−0.58	<.01	0.74	<.01
AWTPi10	−0.11	.11	−0.22	<.01	0.13	.09
%fLDA	−0.49	<.01	−0.42	<.01	0.46	<.01
%pLDA	−0.68	<.01	−0.52	<.01	0.80	<.01

Legend: *AWTPi10* airway wall thickness at an internal perimeter of 10 mm, *ESI* emphysema severity index, *FEV*_*1*_*%* forced expiratory volume in 1 s, *%fLDA* percentage of functional low density area, *FVC* forced vital capacity, *% LAA*_*-950insp*_ percentage of lung attenuation area with values <− 950 Hounsfield Units at inspiratory CT scan, *%LAA*_*-856exp*_ percentage of lung attenuation area with values <− 856 Hounsfield Units at expiratory CT scan, *%pLDA* percentage of persistent low density area

Table 4 displays the mean values of ESI and the functional data of the three groups of patients stratified by quantitative CT (VIDA, Imbio LDA) according to the thresholds to define different degrees of emphysema (NE, ME, SE) and by radiomics (CALIPER) according to the clusters of progressive emphysema severity (G1, G2, G3). Patients allocation differed within the three groups defined by each of the quantitative CT post-processing techniques. However, a significant progressive impairment from patients classified NE or G1 to patients classified SE and G3 was observed. Pulmonary function data were significantly different among the subgroups with various degrees of emphysema with a few exceptions (Table 5). Lower ESI values (< 5) were typical for MEFV curves obtained in COPD patients with NE or ME, whereas higher values (> 5) were observed in patients with SE. Mean of the ESI values differed significantly among the three emphysema groups defined by %LAA_-950insp_ by VIDA *(p* < .001) as well as among the three Imbio LDA groups (*p* < .001) and CALIPER clusters (*p <* .001).

**Table 4:** Relationship among ESI values and functional data across the groups of patients with various degrees of emphysema

Emphysema severity	N	ESI score	FEV_1_%	FVC %	FEV_1_/FVC %	TLC %	RV %	FRC %	DLco %
VIDA									
NE	57	1.1 (1.5)	76.1 (23.6)	93.5 (22.8)	63.1 (8.0)	100.1 (11.8)	115.0 (30.5)	109.3 (18.2)	79.7 (22.9)
ME	58	3.1 (2.6)	63.6 (23.9)	91.4 (21.0)	54.1 (10.2)	106.9 (18.8)	134.1 (51.7)	125.3 (31.0)	75.6 (21.6)
SE	79	6.8 (2.5)	49.9 (23.7)	91.2 (27.8)	42.4 (10.8)	115.2 (15.1)	157.6 (48.2)	147.9 (33.7)	55.9 (19.8)
*ANOVA / Welch’s test p*		***<.001***	***<.001***	***.002***	***<.001***	***<.001***	***<.001***	***<.001***	***<.001***
Imbio LDA									
NE	86	1.5 (1.8)	72.6 (22.9)	92.9 (20.9)	60.5 (9.2)	101.1 (15.6)	118.2 (42.0)	111.5 (23.7)	78.9 (23.2)
ME	46	4.6 (2.7)	63.8 (27.5)	94.1 (25.2)	52.1 (10.1)	111.9 (15.0)	145.6 (47.4)	135.4 (30.5)	72.6 (18.1)
SE	62	7.7 (3.3)	44 (18.8)	88.6 (28.2)	39.6 (9.5)	115.7 (15.5)	160.7 (46.2)	152.1 (32.5)	51.9 (18.9)
*ANOVA / Welch’s test p*		***<.001***	***<.001***	*.442*	***<.001***	***<.001***	***<.001***	***<.001***	***<.001***
CALIPER									
G1	95	1.7 (2.1)	71.9 (23.7)	92.8 (21.9)	59.8 (9.6)	101.2 (14.6)	118.7 (39.4)	113.2 (24.3)	78.4 (23.2)
G2	65	5.4 (2.8)	59.7 (24.9)	97.1 (26.5)	48.8 (11.0)	113.4 (16.5)	145.9 (47.6)	137.9 (30.7)	65.6 (19.0)
G3	34	8.0 (1.7)	36.8 (14.3)	79.5 (23.2)	36.5 (8.0)	118.2 (13.6)	177.3 (44.0)	163.4 (29.9)	48.1 (18.5)
*ANOVA / Welch’s test p*		***<.001***	***<.001***	***.002***	***<.001***	***<.001***	***<.001***	***<.001***	***<.001***

Differences among groups were assessed by analysis of variance and Welch’s tests, expressed in *italics* and in ***bold*** if significant. Values are expressed as mean (SD). *DLco %* percent predicted diffusing lung capacity for carbon monoxide, *FEV*_*1*_*%* percent predicted forced expiratory volume in 1 s, *FRC%* percent predicted functional residual capacity, *FVC%* percent predicted forced vital capacity, *%LAA*_*-950insp*_ percentage of lung attenuation area with values <−950 Hounsfield Units at inspiratory CT scan, ME (moderate emphysema, 6 ≤ %LAA_-950insp_ < 14 if VIDA or 6 ≤ %pLDA < 14 if Imbio LDA), NE (no emphysema, %LAA_-950insp_ < 6 if VIDA or %pLDA < 6 if Imbio LDA), *RV%* percent predicted residual volume, SE (severe emphysema, %LAA_-950insp_ ≥ 14 if VIDA or %LAA_-950insp_ ≥ 14 if Imbio LDA), *TLC%* percent predicted total lung capacity

**Table 5:** Post-hoc analysis of differences between groups with various degrees of emphysema as described in Table 4

		ESI	FEV_1_%	FVC%	FEV_1_/FVC%	TLC%	RV%	FRC%	DLco%
VIDA	p NE/ME	**<.01**	**<.01**	**<.01**	**<.01**	.28	**<.01**	**<.01**	**<.01**
	p NE/SE	**<.01**	**<.01**	**<.01**	**<.01**	**<.01**	**<.01**	**<.01**	**<.01**
	p ME/SE	**<.01**	**<.01**	.52	**<.01**	**<.01**	**<.01**	**<.01**	**<.01**
Imbio LDA	p NE/ME	**<.01**	.16	.96	**<.01**	**<.01**	**<.01**	**<.01**	.21
	p NE/SE	**<.01**	**<.01**	.56	**<.01**	**<.01**	**<.01**	**<.01**	**<.01**
	p ME/SE	**<.01**	**<.01**	.54	**<.01**	.42	.23	**.02**	**<.01**
CALIPER	p G1/G2	**<.01**	**<.01**	.52	**<.01**	**<.01**	**<.01**	**<.01**	**<.01**
	p G2/G3	**<.01**	**<.01**	**<.01**	**<.01**	.28	**<.01**	**<.01**	**<.01**
	p G1/G3	**<.01**	**<.01**	**<.01**	**<.01**	**<.01**	**<.01**	**<.01**	**<.01**

Groups are defined according the classification performed by VIDA and Imbio LDA (NE, ME, SE) and by CALIPER (G1, G2, G3). Differences were analyzed by Games-Howell post-hoc test and *p* values are displayed (significant p values are in bold). Legend: *DLco %* percent predicted diffusing lung capacity for carbon monoxide, *FEV*_*1*_*%* percent predicted forced expiratory volume in 1 s, *FRC%* percent predicted functional residual capacity, *FVC%* percent predicted forced vital capacity, *%LAA*_*-950insp*_ percentage of lung attenuation area with values <−950 Hounsfield Units at inspiratory CT scan, ME (moderate emphysema, 6 ≤ %LAA_-950insp_ < 14 if VIDA or 6 ≤ %pLDA < 14 if Imbio LDA), NE (no emphysema, %LAA_-950insp_ < 6 if VIDA or %pLDA < 6 if Imbio LDA), *RV%* percent predicted residual volume, SE (severe emphysema, %LAA_-950insp_ ≥ 14 if VIDA or %LAA_-950insp_ ≥ 14 if Imbio LDA), *TLC%* percent predicted total lung capacity

As FEV_1_ and FEV_1_/FVC differed significantly between groups with various degrees of emphysema (NE, ME, SE) we performed a ROC analysis (see ROC curves in the Additional file 1). FEV_1_ differentiated NE from ME with 0.62 sensitivity and 0.80 specificity (AUC 0.74) and ME from SE with 0.77 sensitivity and 0.67 specificity (AUC 0.77). Likewise, FEV_1_/FVC differentiated NE from ME with 0.68 sensitivity and 0.88 specificity (AUC 0.83) and ME from SE with 0.82 sensitivity and 0.76 specificity (AUC 0.82).

**Fig. 3:**
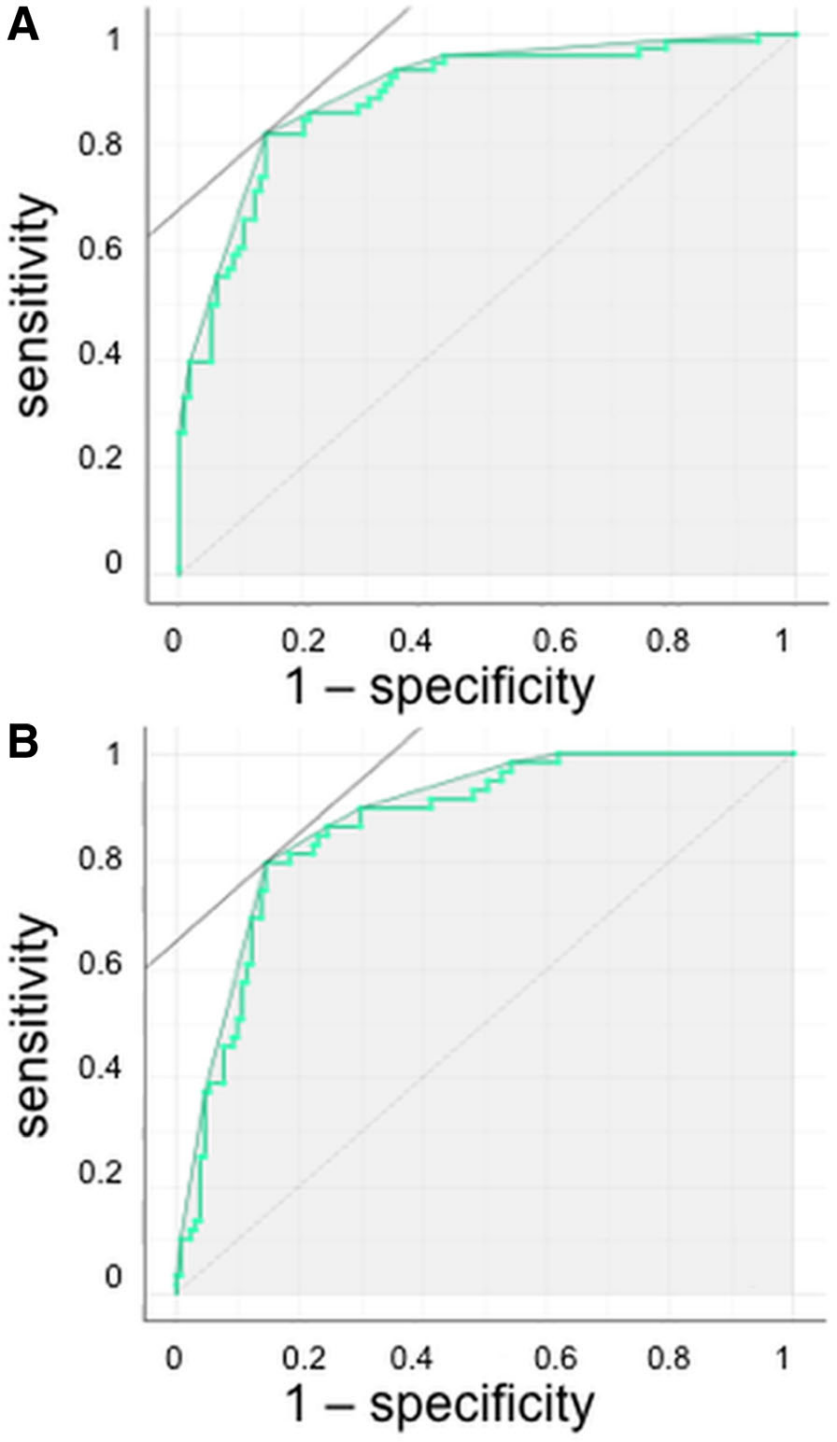
ROC curve over the range of the ESI model output for severe emphysema **a** and no emphysema **b**. Severe emphysema was defined at CT scan as %LAA_-950insp_ ≥ 14 by VIDA whereas no emphysema was defined at CT scan as %LAA_-950insp_ < 6 by VIDA. The total AUC area was of 0.88 for severe emphysema and 0.86 for no emphysema

The graph in Fig. 3a represents the ten folds averaged ROC curve obtained by varying the classification threshold over the range of the logistic regression model output π = Pr (Y = “Severe” | X = ESI) for the probability of “severe” emphysema (%LAA_-950insp_  ≥14, data provided by VIDA). The best results in terms of sensitivity and specificity were relative to the threshold value 0.47, with sensitivity = 0.82 and specificity = 0.87. The total AUC area was of 0.88.

The graph in Fig. 3b represents the ten folds averaged ROC curve obtained by varying the classification threshold over the range of the logistic regression model output π = Pr (Y = “Absence” | X = ESI) for the probability of “absence” of emphysema (%LAA_-950insp_ < 6, data provided by VIDA). The best results in terms of sensitivity and specificity were relative to the threshold value 0.37, with sensitivity = 0.80 and specificity = 0.85. The total AUC area was of 0.86.

Therefore, NE is differentiated from ME/SE with a sensitivity of 0.80 and a specificity of 0.85, whereas SE is differentiated from ME with a sensitivity of 0.82 and a specificity of 0.87 by using the MEFV curve.

**Fig. 4:**
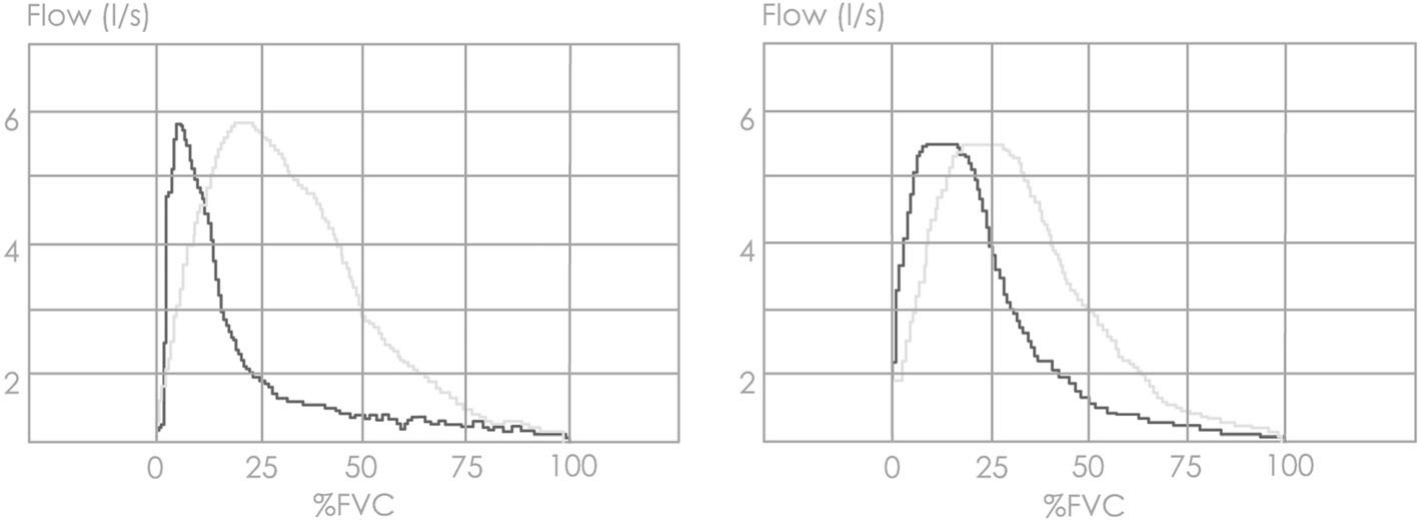
Maximal expiratory flow-volume curves of two representative subjects with severe emphysema or no emphysema. Patient with severe emphysema (*left panel*) had %LAA_-950insp_ = 24 whereas the patient with no emphysema (*right panel*) had %LAA_-950insp_ = 4 at CT. Note the flatter slope in the former when flow was plotted against expired volume (*black lines*) but not pletysmographic thoracic volume (*grey lines*), indicating greater thoracic gas compression at high-to-mid lung volumes

Figure 4 shows differences in MEFV curves of two representative subjects with severe emphysema and no emphysema. The former has a flatter slope when flow is plotted against expired volume but not at pletysmographic thoracic volume, indicating greater thoracic gas compression at high-to-mid lung volumes.

## Discussion

The extensive application of CT scan post-processing techniques has shown that presence and severity of emphysema as assessed by CT does closely reflect lung function presentation in COPD. The main finding of this study is that a mathematical model developed to fit the descending limb of the MEFV curve approximates the multimodality CT-validated emphysema stratification with an accuracy that could be suitable for clinical and research purposes. The model is based only on MEFV curve morphology and, consequently, it is independent from percentage predicted values of pulmonary function.

In recent years quantitative CT enabled radiologists to quantify and localize the relative volumes of emphysema and gas trapping in subjects with COPD by using standard CT metrics of low attenuation areas at pre-determined inspiratory and expiratory X-ray attenuation thresholds [1, 17]. Beside CT quantification of emphysema, the new computational radiomics approach allows to extract multiple features from imaging data and to process them in order to objectively and reproducibly characterize the main pathologic changes in the course of lung diseases. Radiomics artificial intelligence can be used to develop non-invasive imaging biomarkers, which could be helpful in phenotyping heterogeneous diseases, such as COPD [23].

As shown in Table 4, patients allocation in emphysema severity subgroups varies with the different CT metrics and radiomics approaches used. Despite this heterogeneity of classification reflecting the underlying methodological differences of the three CT post-processing analyses, ESI differentiates the progressive severity of emphysema whatever the CT method used to classify patients. CT metrics of emphysema (%LAA_-950insp_ and %pLDA) correlated more strongly to ESI compared to FEV_1_ and FEV_1_/FVC. These results support the usefulness of the MEFV curve in better depicting the emphysema extent as assessed at CT, instead of the standard functional variables (FEV_1_ and FEV_1_/FVC) used in clinical practice.

A recent study from our group showed that a probabilistic model including DLco%, FEV_1_%, FEV_1_/VC, and BMI dissects with accuracy emphysematous from non-emphysematous gas trapping as assessed by standard CT metrics in patients with COPD [9]. Reduction in DLco is considered a marker of emphysema in subjects with COPD [24]. However, measurement of DLco is not widely performed and standard pulmonary function parameters have a wide inter-individual variability, even after normalization for ethnicity, age, and body size [10]. In the current study we overcame these limitations by a model depending only from MEFV curve morphology that does not require normalization by reference equations and, being a mathematical model and not a probabilistic one, it does not depend from regression coefficients reflecting the characteristics of a training set.

A role for the MEFV curve in predicting the risk of emphysema was introduced as early as 1976 by Saltzman et al. [25]. They proposed that a kinging of the descending limb of the MEFV curve might represent a sign of airway collapse reflecting the presence of emphysema [25]. More recently the angle between two regression lines fitted to the descending limb of the MEFV curve resulted to be predictive of the presence of emphysema on CT scan with good specificity but low sensitivity [26].

The kinking of MEFV curve in emphysema can be interpreted on the grounds of the wave-speed theory [27]. During forced expiration, alveolar pressure increases and gas is compressed within the lung, thus reducing lung volume and elastic recoil pressure. As a result, driving pressure and distending pressure at choke point decrease, thereby reducing maximal flow. As demonstrated in a recent study, this effect may be magnified in emphysema because of the abrupt fall of lung elastic recoil at high lung volumes and the larger amount of gas to be compressed [28]. The composite result of these physical phenomena is a flattening the MEFV curve. By contrast, the scooping of MEFV curve in COPD patients with predominant conductive airway disease may reflect a smooth decrease of lung elastic recoil and a transition of the choke point towards the lung periphery with less gas compression [29]. Mechanisms that may contribute to the kinking of forced expiratory flow in emphysema are a sudden airway narrowing due to sharp decrease in lung elastic recoil and a sharp decrease of thoracic gas compression from high-to-mid lung volumes [30]. Therefore, flattening of the MEFV curve from high-to-mid lung volumes (Fig. 4) could be a predictor of emphysema. At variance with the above quoted paper on the analysis of the MEFV curve [26], the model presented here predicts presence and severity of emphysema with a considerable level of accuracy.

This study has some limitations. First, it included a relatively small number of white Caucasian subjects that cannot be considered representative of the wide clinical spectrum of COPD in the general population. However, the distribution of CT metrics and their average values were similar to those reported from the COPDGene Study that included more than 10,000 subjects of two different ethnicities [9, 31]. Second, the CT scans were acquired without spirometric control of lung inflation level during the acquisition. However, all subjects received prior cautious instruction on how to perform the respiratory maneuvers just before undergoing CT scanning by dedicated personnel. Third, radiomics is very sensitive to protocol acquisition parameters, algorithm definitions, and image processing [23]. Lack of standardization of these components severely hampers reproducibility and comparability of results. In this study all CT scans were acquired with the same protocol by the same personnel in each study center, and after calibration of CT scanner before each examination. Fourth, models based on MEFV curve strictly depend on patient effort during spirometry, as it affects the magnitude of thoracic gas compression [32]. This is a major determinant of the shape of the MEFV curve, particularly in subjects with predominant emphysema [13]. However, accurate technicians training could overcome this limitation. Fifth, emphysema CT subtypes were not evaluated in this study. Assessment of ESI in each emphysema subtype would be an important subject for future studies, in particular to demonstrate if any specific subtype could be predicted more accurately than others.

## Conclusions

This study demonstrates that the presence of emphysema and its severity in patients with COPD, as defined on inspiratory-expiratory CT scan by standard metrics and co-registration analysis, as well as by a computational unsupervised CT-based radiomics, can be accurately estimated by a mathematical model based on MEFV curve morphology. The model is independent from reference values and, if confirmed in larger populations of patients with COPD, it could be helpful in clinical practice to personalize therapy, to select patients for clinical and pharmacologic trials, and for the interpretation of their results whenever spirometry is the only available examination.

## Additional file

Additional file 1: Theoretical background and description of ESI method (PDF 499 kb)

## Abbreviations

%fLDA: Percentage of functional low density area
%LAA_-856exp_: Percentage of lung attenuation area with values <− 856 Hounsfield Units at inspiratory CT scan
%LAA_-950insp_: Percentage of lung attenuation area with values <− 950 Hounsfield Units at inspiratory CT scan
%pLDA: Percentage of persistent low density area
AWTPi10: Airway wall thickness at an internal perimeter of 10 mm
BMI: Body mass index
COPD: Chronic obstructive pulmonary disease
CT: Computed tomography
DLco%: Percent predicted diffusing lung capacity for carbon monoxide
ESI: Emphysema Severity Index
FEV_1_%: Percent predicted forced expiratory volume in 1 s
FRC%: Percent predicted functional residual capacity
FVC%: Percent predicted forced vital capacity
LAA: Low Attenuation Area
ME: Moderate emphysema
MEFV: Maximum expiratory flow-volume
NE: No emphysema
RV%: Percent predicted residual volume
SE: Severe emphysema
TLC%: Percent predicted total lung capacity
VC: Vital capacity
