# Supplementary material for: Spirometric assessment of emphysema presence and severity as measured by quantitative CT and CT-based radiomics in COPD
Source: Respir Res. 2019 May 23;20:101. doi: 10.1186/s12931-019-1049-3 (PMC6533715; doi:10.1186/s12931-019-1049-3)
Supplement: Supplementary file 1 — Theoretical background and description of ESI method (PDF 499 kb) [file 12931_2019_1049_MOESM1_ESM.pdf]

Supplemental Material: *Theoretical background and description of ESI method*

In the past many studies in human subjects have shown the presence of a functional association between the airflow at mouth, the instantaneous volume of the lung, and the pressure applied on the surface of the lung. [1-3] In particular the analysis of the maximum expiratory flow-volume (MEFV) curve has been used for many years to characterize the functional behavior of the bronchial tree and the surrounding parenchyma.

In this study we tested a parametric biomechanical model representing a theoretical approximation of the shape of the descending limb of the MEFV curve to assess the severity of emphysema in patients with COPD by spirometry. The Emphsyema Severity Index (ESI) application software is based on a mathematical model developed to approximate the MEFV curve of each subject to ultimately provide a quantitative score ranging from 1 to 10. The ESI score represents a practical application of a biomechanical model developed a priori. No retrospective statistical inference or standardization of input parameters was required.

The main physical principle inspiring this approach is that the pressure lost (*Pl*) at a given time *t* along an airway segment could be considered proportional to a specific friction factor (*Ff*), the air density (*d*) and the air velocity (*v*), similarly to the theory of the circular ducts. An inverse relationship between the pressure lost and the mean diameter (*D*) of the airway is also supposed obtaining the following equation (better known as Darcy equation):

$$Pl = \frac{Ff \cdot d \cdot v^2}{2 \cdot D} \quad (1)$$

By considering the hypothesis of laminar flow, the calculus of the friction factor depends uniquely on the Reynolds number *Rn*

$$Ff = \frac{k1}{Rn} \quad (2) \text{ where } \quad Rn = \frac{v \cdot D}{vis} \quad (3)$$

k1 is a constant and the variable *vis* is the dynamic viscosity of the air fluid.

Substituting equations (2) and (3) into equation (1) we obtain the pressure drop in the case of laminar flow as:

$$Pl = \frac{k1/2 \cdot vis \cdot d \cdot v}{D^2} \quad (5)$$

Considering a circular airway section we can write the air velocity in the segment as:

$$v = \frac{4 \cdot \Phi}{D^2 \cdot \pi} \quad (6)$$

where Φ is the resulting flow and D is the mean inner diameter of the airway segment. Substituting equation (6) into (5) we obtain the association between the airflow and the pressure lost, as of laminar flow hypothesis.

$$Pl = \frac{k2 \cdot vis \cdot d \cdot \Phi}{D^4} \quad (7)$$

Equation (7) shows that the relationship between the pressure lost and the airflow seems to be linear in laminar flow approximation.

In the past some authors proposed lumped parametric models to fit the MEFV curve acquired during maximal effort test, taking into account linear association profiles between airways resistance and airflow. The studies obtained acceptable waveforms and good fitting of the curve. [3]

At variance with past models, we proposed a mathematical model for the approximation of the MEFV curve developed under the hypothesis that the airflow measured at the mouth through standard spirometry could not be considered as a laminar flow. As a consequence the *Ff* variable could not be calculated as a scaled inverse of the Reynolds number *Rn* (laminar flow), but a more complex calculation is required to estimate the pressure drop. Colebrook proposed the following equation for the calculation of the friction factor, where *Ru* is the mean relative roughness of the airway segment [4]:

$$\frac{1}{Ff^{0.5}} = -2 \cdot log10\left(\frac{Ru}{3.7 \cdot D} + \frac{2,51}{Rn \cdot Ff^{0.5}}\right) \quad (8)$$

Although the Colebrook's equation is usually solved numerically due to its implicit nature, simplified versions have been proposed in the past (i.e. the Altshul-Tsal [4] formulation to estimate an approximated *Ff* factor). Nonetheless, despite of the analytical procedure for the estimation of *Ff,* the final equation that describes the pressure drop is the following:

$$Pl = \frac{K3 \cdot Ff \cdot d \cdot \Phi^2}{D^5} \quad (9)$$

where *K3* and *Ff* are constant values for a specific patient and airway segment, *d* is the air density, *Φ* is the resulting flow and *D* is the mean inner diameter.

One interesting observation deriving from these mathematical models is that the pressure drop *Pl* along a segment is inversely proportional to the diameter of the airway power 4 or 5. It follows that a minimal

variation in airway diameter is amplified to cause a significant pressure drop along the whole segment. This shows how the regulation of the respiratory airways walls efficiently modulates the airflow.

Another important characteristic of the non-laminar flow hypothesis is the quadratic association between the airflow and the pressure lost along the airways, while this relationship would be linear if the measured flow at the mouth was hypothesized as laminar.

As a consequence the lumped parametric model of a maximal flow volume curve implemented in the ESI model is based on the following non-linear equation:

$$\Phi(V) = \frac{1 - a1 \cdot V^2}{a2 + a3 \cdot V^2} \quad (10)$$

where the numerator represents a simple model of the quadratic pressure profile during a maximal function test and the denominator represents the model of the exhaled volume-dependent quadratic airways resistance profiles. The best *a1*, *a2* and *a3* coefficients are estimated iteratively by the software, which performs a Least Mean Squares fitting of the descending limb of the MEFV curve.

Once the best mathematical model fitting the raw data is obtained, the software then proceeds to the calculus of the first and second derivatives for the specific fitted MEFV curve. This procedure is performed to search the inflection point *Vf* of the descending limb of the MEFV curve.

$$\frac{\partial'' \Phi(V)}{\partial V} = 0 \quad (11)$$

The point *Vf* is very important in assessing emphysema severity, as its abscissa represents the lung volume (in liters) at which the resistance profile becomes influenced by the lost in elastic recoil due to

the parenchymal destruction. This creates an inversion in the concavity of the descending limb of the MEFV curve.

This type of analysis was not possible before because of the hypothesis of the above-mentioned linear models. Indeed, in those models the second derivatives profiles did not admit acceptable solutions to the equation (11).

After the solutions of equation (11) are calculated, the first derivative is then evaluated in correspondence of the acceptable solution (volume value) Vf and re-mapped between 0-10 over the angle range *(20° - 80°)* generating the quantitative index called ESI. The computation does not require standardization of input parameters, as it is directly related to the shape of the curve. Therefore, ESI is independent from percentage predicted values of pulmonary function variables. ESI value was computed in each patient using a specifically developed software application, based on the above theoretical basis. A numerical output value ranging from 0 to 10 was used to stratify the dataset of 194 COPD patients according to the estimated emphysema severity.

The Figure 1S shows a box-plot representing the $\%LAA_{-950insp}$ distribution (I-III quartiles) in the validation dataset in three subgroups: no emphysema (NE, $\%LAA_{-950insp} < 6$), moderate emphysema (ME, $6 \leq \%LAA_{-950insp} < 14$), and severe emphysema (SE, $\%LAA_{-950insp} \geq 14$). The intersection of the ESI values (*red line*) with the corresponding box indicates the most probable severity class for the case analyzed. In this example the case analyzed had a low probability of having SE.

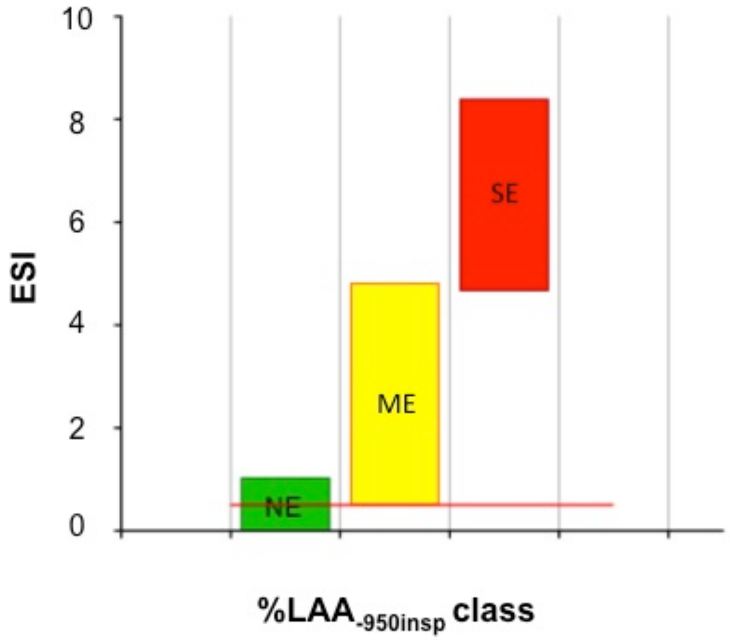

As FEV_1 and FEV_1/FVC differed significantly between groups with various degrees of emphysema (NE, ME, SE) we performed a ROC analysis. FEV_1 differentiated NE from ME with 0.62 sensitivity and 0.80 specificity (AUC 0.74, Figure 2S) and ME from SE with 0.77 sensitivity and 0.67 specificity (AUC 0.77, Figure 3S). Likewise, FEV_1/FVC differentiated NE from ME with 0.68 sensitivity and 0.88 specificity (AUC 0.83, Figure 4S) and ME from SE with 0.82 sensitivity and 0.76 specificity (AUC 0.82, Figure 5S). Accuracies and ROC curves of the ESI model to differentiate the groups with different degrees of emphysema are reported in the main text.

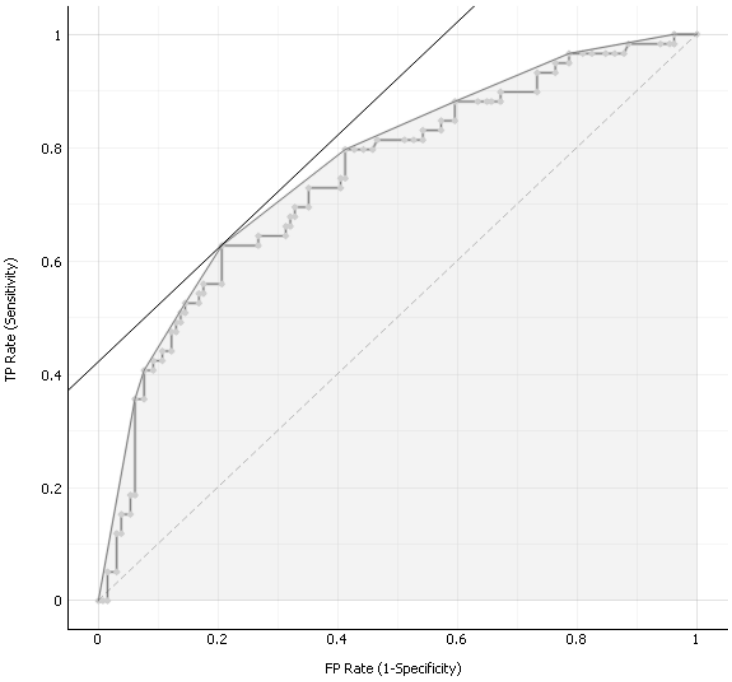

Figure 2S. ROC curve over the range of FEV_1 to differentiate NE from ME. AUC 0.74, 0.62 sensitivity and 0.80 specificity.

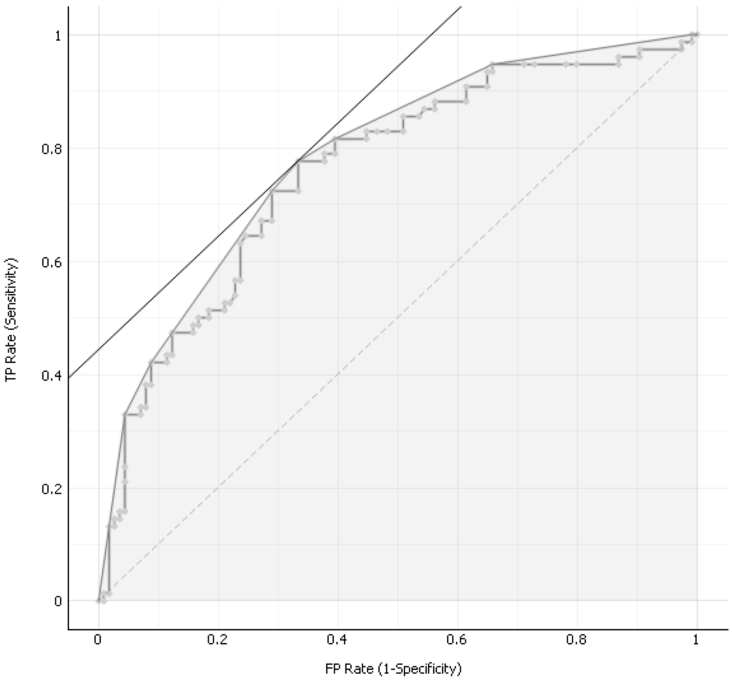

Figure 3S. ROC curve over the range of FEV_1 to differentiate ME from SE. AUC 0.77, 0.77 sensitivity and 0.67 specificity.

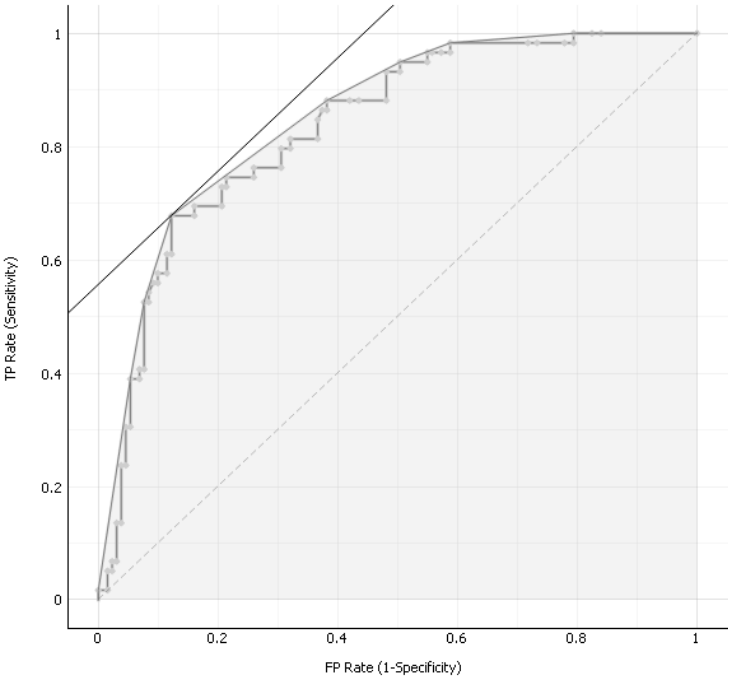

Figure 4S. ROC curve over the range of FEV_1/FVC to differentiate NE from ME. AUC 0.83, 0.68 sensitivity and 0.88 specificity.

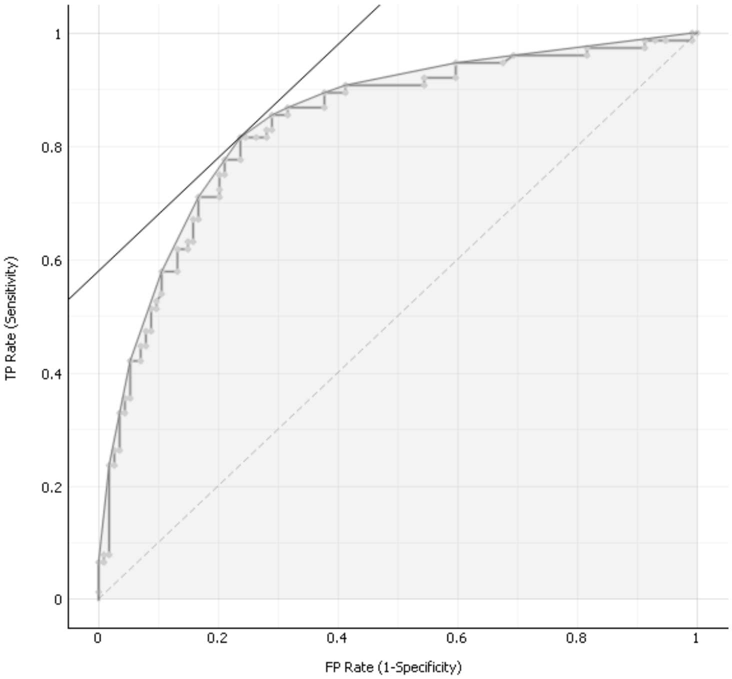

Figure 5S. ROC curve over the range of FEV_1/FVC to differentiate ME from SE. AUC 0.82, 0.82 sensitivity and 0.76 specificity.

References

1. Fry DL. Theoretical considerations of the bronchial pressure-flow-volume relationships with particular reference to the maximum expiratory flow volume curve. Phys Med Biol. 1958; 3:174-94.

2. Abboud S, Barnea O, Guber A, Narkiss N, Bruderman I. Maximum expiratory flow-volume curve: mathematical model and experimental results. Med Eng Phys. 1995; 17:332-6.

3. Zheng CJ, Adams AB, McGrail MP, Marini JJ, Greaves IA. A proposed curvilinearity index for quantifying airflow obstruction. Respir Care. 2006; 51:40-5.

4. Hughes WF. Theory and problems of fluid dynamics. New York: Shaum; 1967.
